# G9a participates in nerve injury-induced Kcna2 downregulation in primary sensory neurons

**DOI:** 10.1038/srep37704

**Published:** 2016-11-22

**Authors:** Lingli Liang, Xiyao Gu, Jian-Yuan Zhao, Shaogen Wu, Xuerong Miao, Jifang Xiao, Kai Mo, Jun Zhang, Brianna Marie Lutz, Alex Bekker, Yuan-Xiang Tao

**Affiliations:** 1 Department of Anesthesiology, New Jersey Medical School, Rutgers, The State University of New Jersey, Newark, NJ, USA; 2 State Key Laboratory of Genetic Engineering, Collaborative Innovation Center for Genetics and Development, School of Life Sciences, Fudan University, Shanghai, China; 3 Departments of Cell Biology & Molecular Medicine and Physiology, Pharmacology & Neuroscience, New Jersey Medical School, Rutgers, The State University of New Jersey, Newark, NJ, USA

## Abstract

Nerve injury-induced downregulation of voltage-gated potassium channel subunit Kcna2 in the dorsal root ganglion (DRG) is critical for DRG neuronal excitability and neuropathic pain genesis. However, how nerve injury causes this downregulation is still elusive. Euchromatic histone-lysine N-methyltransferase 2, also known as G9a, methylates histone H3 on lysine residue 9 to predominantly produce a dynamic histone dimethylation, resulting in condensed chromatin and gene transcriptional repression. We showed here that blocking nerve injury-induced increase in G9a rescued Kcna2 mRNA and protein expression in the axotomized DRG and attenuated the development of nerve injury-induced pain hypersensitivity. Mimicking this increase decreased Kcna2 mRNA and protein expression, reduced Kv current, and increased excitability in the DRG neurons and led to spinal cord central sensitization and neuropathic pain-like symptoms. G9a mRNA is co-localized with Kcna2 mRNA in the DRG neurons. These findings indicate that G9a contributes to neuropathic pain development through epigenetic silencing of Kcna2 in the axotomized DRG.

Neuropathic pain is one of major clinical diseases characterized by spontaneous ongoing or intermittent burning pain, allodynia, and hyperalgesia. It is caused by trauma (e.g., peripheral nerve, spinal cord, or brain injury) and some disorders (e.g., multiple sclerosis, stroke, human immunodeficiency virus-induced neuropathy, and diabetes)^1^. Therapeutic approaches of neuropathic pain management provide symptomatic relief, but most of these approaches are nonspecific in regards to the cause of this disorder and often provide unsatisfactory relief^2^. Peripheral nerve injury leads to abnormal ectopic firing in the neuromas at the injured site and dorsal root ganglion (DRG) neurons^1^,^3^. This ectopic firing is thought to induce neuropathic pain. Therefore, understanding how abnormal neuronal activities arise in the DRG neurons may provide new and specific therapeutic strategies in neuropathic pain management.

Voltage-gated potassium channel subunit Kcna2 belongs to a family of delayed rectifiers, which control the excitability of DRG neurons by allowing neurons to efficiently repolarize following an action potential. Approximately 70% of DRG neurons are positive for Kcna2, most of which are medium and large in size^4^,^5^. Peripheral nerve injury dramatically decreased the expression of Kcna2 mRNA and protein in the axotomized DRG^4^,^5^,^6^,^7^,^8^,^9^,^10^. This decrease is responsible for neuropathic pain development as rescuing Kcna2 expression in the axotomized DRG attenuated nerve injury-induced pain hypersensitivity^4^. Moreover, mimicking this decrease reduced total Kv current, depolarized the resting membrane potential, diminished current threshold for action potential (AP) activation, and led to pain hypersensitivity^10^. We previously reported that an endogenous Kcna2 antisense (AS) RNA, a long non-coding RNA, is a trigger in nerve injury-induced DRG Kcna2 downregulation under neuropathic pain conditions^10^,^11^,^12^. However, blocking increased Kcna2 AS RNA only partially rescued Kcna2 mRNA and protein expression in the axotomized DRG neurons^10^. This indicates that other mechanisms may also participate in DRG Kcna2 downregulation following peripheral nerve injury.

G9a, a histone methyltransferase, methylates histone H3 on lysine residue 9 (H3K9) to produce dimethylation (H3K9me2), a dynamic histone methylation mark^13^. This modification results in condensed chromatin and gene transcriptional repression^14^. Although a recent study showed an involvement of G9a in nerve injury-induced downregulation of some DRG potassium channel genes^15^, whether and how G9a regulates Kcna2, a highly expressed potassium channel in the DRG^4^,^5^, under neuropathic pain conditions is unknown. Here, we report that peripheral nerve injury increases the expression of G9a and H3K9me2 in the axotomized DRG neurons. These increases contribute to neuropathic pain development through epigenetic silencing of DRG Kcna2.

## Results

### G9a and H3K9me2 are increased in the axotomized DRG neurons after nerve injury

**Figure 1:**
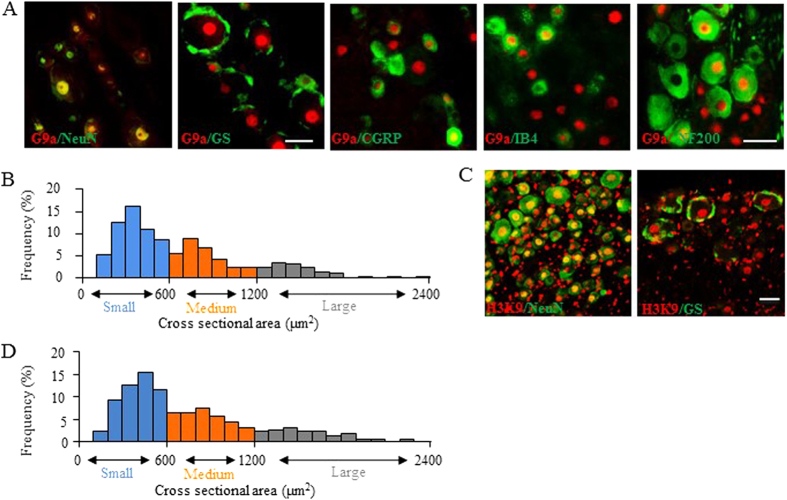
Cellular distribution of G9a and H3K9me2 in dorsal root ganglion (DRG). n = 3 mice. (**A**) Representative examples showing that G9a is co-expressed exclusively with NeuN in cellular nuclei, undetected in glutamine synthetase (GS)-labeled cells, and distributed in calcitonin gene-related peptide (CGRP)-, isolectin B4 (IB4)-, or neurofilament 200 (NF200)-positive neurons in DRG. Scale bars: 25 μm for NeuN and GS and 50 μm for CGRP, IB4, and NF200. (**B**) Histogram showing distribution of G9a-labeled neuronal somata in DRG. Small: 59%. Medium: 28%. Large: 13%. (**C**) Representative examples showing that H3K9me2 is co-expressed with NeuN in cellular nuclei and detected in GS-positive satellite cells. Scale bars: 50 μm. (**D**) Histogram showing distribution of H3K9me2-labeled neuronal somata in DRG. Small: 57%. Medium: 29%. Large: 14%.

To examine whether G9a regulates Kcna2 expression in DRG, we first analyzed the distribution pattern of G9a in the DRG by double immunohistochemistry for G9a and NeuN (a specific neuronal marker) or glutamine synthetase (GS, a marker for satellite glial cells). G9a co-expressed with NeuN in cellular nuclei and was not detected in GS-labeled cells (Fig. 1A). Approximately 12% of DRG neurons (120 of 989) were labeled for G9a, of which about 31% of the G9a-labeled neurons were positive for calcitonin gene-related peptide (CGRP, a marker for small DRG peptidergic neurons), 29% for isolectin B4 (IB4, a marker for small non-peptidergic neurons), and 43% for neurofilament-200 (NF200, a marker for medium/large cells and myelinated A-fibers) (Fig. 1A). Consistently, the cross sectional area analysis of neuronal somata displayed that approximately 59% of G9a-labelled neurons are small (<600 μm^2^ in area), 28% medium (600–1,200 μm^2^ in area), and 13% large (>1,200 μm^2^ in area) (Fig. 1B). However, H3K9me2 was detected in both NeuN- and GS-labeled DRG cells (Fig. 1C). Approximately 34% of DRG neurons (358 of 1054) were labeled for H3K9me2. The cross sectional area analysis showed that approximately 57% of H3K9me2-positive neurons are small (<600 μm^2^ in area), 29% medium (600–1,200 μm^2^ in area), and 14% large (>1,200 μm^2^ in area) (Fig. 1D).

**Figure 2:**
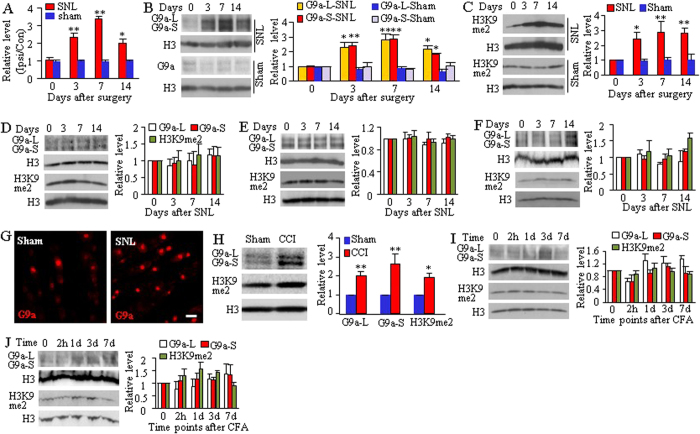
Nerve injury-induced increase in euchromatic histone-lysine N-methyltransferase 2 *(Ehmt2)* mRNA, G9a’s two protein isoforms and H3K9me2 in the axotomized DRG neurons. (**A**) *Ehmt2* mRNA expression in the ipsilateral (Ipsi) L4 (axotomized) DRG after SNL or sham surgery. Con: contralateral. n = 12 mice/time point/group. *P < 0.05 or **P < 0.01 *vs* the corresponding control group (0 day), one-way ANOVA followed by post hoc Tukey test. (**B**,**C**) Expression of G9a’s two protein isoforms (**B**) and H3K9me2 (**C**) in the ipsilateral L4 (axotomized) DRG after SNL or sham surgery. Representative Western blots (left panels) and a summary of densitometric analysis (right graphs) are shown. L: long isoform. S: short isoform. n = 6 mice/time point/group. *P < 0.05 or **P < 0.01 *vs* the corresponding control group (0 day), one-way ANOVA followed by post hoc Tukey test. Full-length blots are presented in Supplemental Figure 1. (**D**–**F**) Expression of two G9a isoforms and H3K9me2 in the ipsilateral L3 (intact) DRG (**D**), contralateral L4 DRG (**E**) and ipsilateral L4 spinal cord (**F**) after SNL. Representative Western blots (left panels) and a summary of densitometric analysis (right graphs) are shown. n = 6 mice/time point. Full-length blots are presented in Supplemental Figure 1. (**G**) Representative examples showing number of G9a-labeled cellular nuclei in the ipsilateral L4 DRG on day 7 post-SNL or sham surgery. Scale bar: 50 μm. (**H**) Expression of G9a’s two protein isoforms and H3K9me2 in the ipsilateral L3/4 DRGs on day 7 after CCI or sham surgery. n = 3 mice/group. *P < 0.05 or **P < 0.01 *vs* the corresponding sham group by two-tailed paired t-test. Full-length blots are presented in Supplemental Figure 1. (**I**,**J**) Expression of two G9a isoforms and H3K9me2 in the L3/4 DRGs (**I**) and L3/4 spinal cord (**J**) on the ipsilateral side after CFA injection. Representative Western blots (left panels) and a summary of densitometric analysis (right graphs) are shown. n = 6 mice/time point. Full-length blots are presented in Supplemental Figure 1.

We further examined whether the activity and expression of G9a were altered in DRG following peripheral nerve injury. A preclinical mouse model of unilateral fourth lumbar (L4) spinal nerve ligation (SNL)^16^ was carried out. The levels of euchromatic histone-lysine N-methyltransferase 2 (*Ehmt2*) mRNA (encoding G9a protein, Fig. 2A) and the amounts of G9a’s two protein isoforms (including the 165 kDa long isoform and 140 kDa short isoform; Fig. 2B) were time-dependently increased in the ipsilateral L4 (axotomized) DRG on days 3, 7, and 14 post-SNL, but not after sham surgery. SNL also led to a time-dependent increase in the level of H3K9me2 in the ipsilateral L4 DRG (Fig. 2C). Neither SNL nor sham surgery altered basal expression of G9a’s two protein isoforms or H3K9me2 in the contralateral L4 DRG, the ipsilateral L3 (intact) DRG, and ipsilateral L4 spinal cord (Fig. 2D–F). Immunofluorescent staining showed that the numbers of G9a-labeled neurons in the ipsilateral L4 DRG on day 7 post-SNL were increased by 2.7-fold as compared to the corresponding sham groups (Fig. 2G). We also observed the increased G9a expression in the axotomized DRGs after chronic constriction injury of sciatic nerve (CCI; Fig. 2H), another preclinical neuropathic pain model^17^. The levels of G9a’s two protein isoforms and H3K9me2 on day 7 post-CCI were higher than those on day 7 post-sham surgery (Fig. 2H). To test whether these increases were a specific response to peripheral nerve injury, we injected complete Freund’s adjuvant (CFA) into the plantar side of one hindpaw to induce peripheral inflammation in mice^18^,^19^. The amounts of G9a and H3K9me2 proteins were not significantly altered in either L3/4 DRGs (Fig. 2I) or L3/4 spinal cord (Fig. 2J) on the ipsilateral side from 2 h to 7 days post-CFA. Our findings indicate that induction of G9a expression and increased level of H3K9me2 occurs exclusively in the axotomized DRG neurons and specifically in response to peripheral nerve injury.

### Blocking increased G9a in the axotomized DRG attenuates neuropathic pain

**Figure 3:**
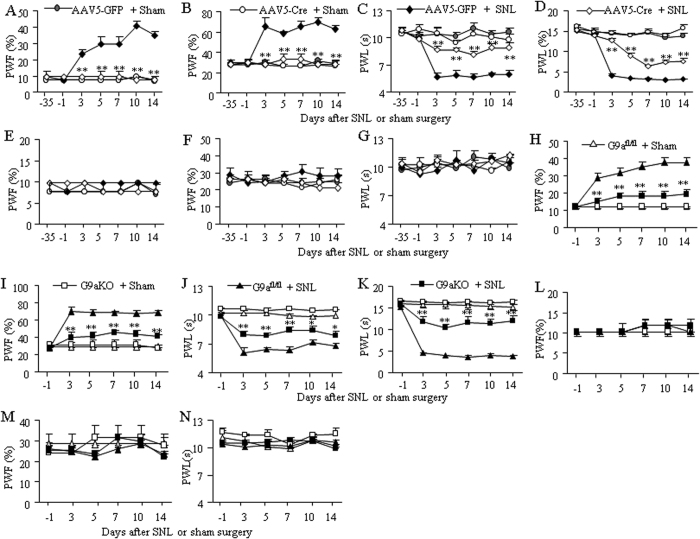
Blocking increased DRG G9a mitigates neuropathic pain. (**A**–**G**) Paw withdrawal frequency (PWF) to low (0.07 g; **A**,**E**) and median (0.4 g; **B**,**F**) force von Frey filament stimuli and paw withdrawal latency (PWL) to thermal (**C**,**G**) and cold (**D**) stimuli on the ipsilateral side (**A**–**D**) and contralateral side (**E**–**G**) of G9a^fl/fl^ mice with microinjection of AAV5-GFP or AAV5-Cre into the ipsilateral L4 DRG post-SNL or sham surgery. n = 7 mice/group. *P* < 0.05 or **P < 0.01 *vs* the AAV5-GFP-injected SNL mice at the corresponding time point, two-way ANOVA followed by post hoc Tukey test. (**H**–**N**) Paw withdrawal frequency (PWF) to low (0.07 g; **H**,**L**) and median (0.4 g; **I**,**M**) force von Frey filament stimuli and paw withdrawal latency (PWL) to thermal (**J**,**N**) and cold (**K**) stimuli on the ipsilateral (**H**–**K**) and contralateral (**L**–**N**) sides of G9a^fl/fl^ mice or G9a conditional knockout (G9aKO) mice after SNL or sham surgery. n = 7 mice/group. *P < 0.05 or **P < 0.01 *vs* the SNL G9a^fl/fl^ mice at the corresponding time point, two-way ANOVA followed by post hoc Tukey test.

To examine if increased G9a in the axotomized DRG has a functional effect on neuropathic pain, we next observed whether blocking increased G9a in the axotomized DRG through microinjection of AAV5-Cre into the ipsilateral L4 DRG of G9a^fl/fl^ mice affected SNL-induced pain hypersensitivity. AAV5-GFP was used as a control. Mice were subjected to SNL or sham surgery 5 weeks after viral microinjection. Basal paw withdrawal responses to mechanical, thermal, and cold stimuli were similar between the two virus-injected groups (Fig. 3A–G). As expected, SNL led to mechanical allodynia and thermal or cold hyperalgesia on the ipsilateral (but not contralateral) side of the AAV5-GFP-injected group from days 3 to 14 after SNL (Fig. 3A–D). These pain hypersensitivities were abolished or ameliorated on the ipsilateral side of the AAV5-Cre-injected group (Fig. 3A–D). Paw withdrawal frequency to mechanical stimulation did not change compared to basal values and paw withdrawal latencies to thermal or cold stimulation were higher compared to the AAV5-GFP-injected mice during the observation period (Fig. 3A–D). DRG microinjection may cause cell damage although the injected DRGs retained their structural integrity and displayed no significant changes in the numbers of neurons or satellite cells (data not shown). To exclude the possibility that the observed effects above were produced by DRG microinjection, we examined SNL-induced pain hypersensitivity in the conditional G9a knockout (G9aKO) mice. Like AAV5-Cre-injected G9a^fl/fl^ mice, G9aKO mice exhibited the reduced paw withdrawal frequency to mechanical stimulation and increased paw withdrawal latency to thermal or cold stimulation compared to G9a^fl/fl^ mice on the ipsilateral side from days 3 to 14 after SNL (Fig. 3H–K). Also, mechanical, thermal, or cold stimuli-induced responses did not change in sham virus-injected and sham genetic KO mice (Fig. 3A–N). Collectively, our findings indicate that G9a in the axotomized DRG may be required for neuropathic pain induction.

### DRG G9a overexpression induced pain hypersensitivities

**Figure 4:**
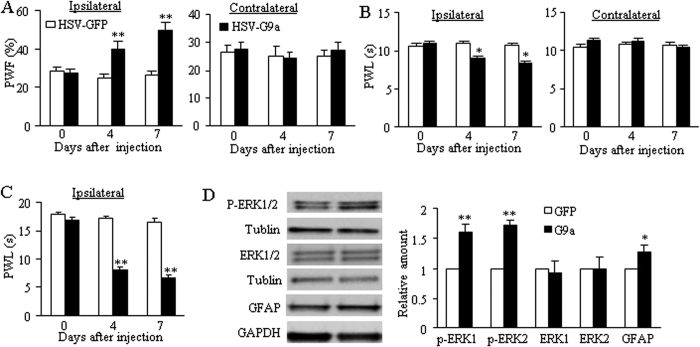
DRG G9a overexpression produces neuropathic pain-like behaviors. Paw withdrawal frequency (PWF) to mechanical stimulation (0.4 g; **A**) and paw withdrawal latency (PWL) to thermal (**B**) and cold (**C**) stimuli on the ipsilateral and contralateral sides from the HSV-GFP-injected group and the HSV-G9a-injected group. n = 7–8 mice/group. *P < 0.05 or **P < 0.01 *vs* the corresponding baseline (0 day), two-way ANOVA followed by post hoc Tukey test. (**D**) Left panel showed the typical picture for increased p-ERK1/2 and GFAP expression in the ipsilateral L3/4 spinal dorsal horn from mice on day 7 after microinjection of HSV-GFP or HSV-G9a into unilateral L3/4 DRGs. Right panel showed statistical analysis of p-ERK1/2, ERK1/2 and GFAP. n = 6 mice/group. *P < 0.05 or **P < 0.01 *vs* the HSV-GFP-injected group by two-tailed paired t-test. Full-length blots are presented in Supplemental Figure 1.

To further examine whether the increased G9a in the DRG was sufficient for neuropathic pain induction, we microinjected HSV that expressed full-length G9a protein (HSV-G9a) into unilateral L3/4 DRGs of naïve adult mice. HSV-GFP was used as a control. Mice injected with HSV-G9a, but not HSV-GFP, exhibited significant increases in paw withdrawal frequencies in response to mechanical stimuli and marked decreases in paw withdrawal latencies in response to thermal and cold stimuli, respectively, on the ipsilateral side (Fig. 4A–C). These alterations occurred 4 days post-injection and persisted for at least 7 days post-injection (Fig. 4A–C). Neither virus affected locomotor activity (data not shown) or basal contralateral paw withdrawal responses (Fig. 4A,B). These findings indicate that the increased G9a in DRG leads to mechanical allodynia and thermal and cold hyperalgesia.

We also asked whether increased G9a in the DRG altered spinal centralization. The levels of phosphorylated extracellular signal-regulated kinase 1/2 (p-ERK1/2, a marker for neuronal hyperactivation) and glial fibrillary acidic protein (GFAP, a marker for astrocyte hyperactivation) significantly increased in the ipsilateral L3/4 dorsal horn of spinal cord on day 7 after microinjection of HSV-G9a compared to those after microinjection of HSV-GFP (Fig. 4D). These findings further support our behavioral observations described above.

### G9a regulation of Kcna2 expression in the axotomized DRG following SNL

**Figure 5:**
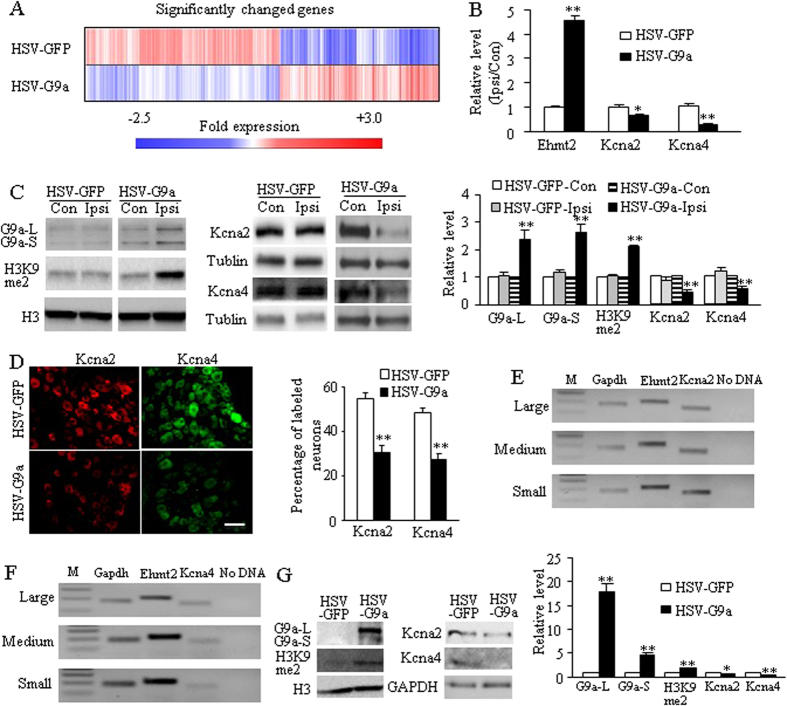
G9a regulation of Kcna2 and Kcna4 expression in the DRG. (**A**) Heat map showing the downregulated genes (blue) and the upregulated genes (red) in the ipsilateral L3/4 DRGs 7 days after microinjection of HSV-G9a or HSV-GFP demonstrated by microarray analysis. (**B**,**C**) The amounts of *Ehmt2*, *Kcna2*, and *Kcna4* mRNAs (**B**) and the levels of G9a’s two protein isoforms, H3K9me2, Kcna2, and Kcna4 (**C**) in the ipsilateral (Ipsi) and contralateral (Con) L3/4 DRGs 7 days after microinjection of HSV-G9a or HSV-GFP. n = 12 mice/group for RT-PCR and 6 mice/group for Western blot. *P < 0.05 or **P < 0.01 *vs* the corresponding HSV-GFP-treated group or the corresponding contralateral side, two-tailed paired t-test for 2 groups, one-way ANOVA followed by post hoc Tukey test for 4 groups. Full-length blots are presented in Supplemental Figure 1. (**D**) Numbers of Kcna2- and Kcna4-labeled neurons in the ipsilateral L4 DRG 7 days after microinjection of HSV-G9a or HSV-GFP. Representative immunohistochemical images (left) and a summary of analysis on the number of Kv1.2- and Kv1.4-labeled neurons (Right) are shown. n = 3 mice/group. **P < 0.01 *vs* the corresponding HSV-GFP-injected group by two-tailed paired t-test. Scale bar: 25 μm. (**E**,**F**) Co-expression of *Ehmt2* mRNA with *Kcna2* mRNA (**E**), and *Kcna4* mRNA (**F**) in individual small, medium and large DRG neurons. *Gapdh* mRNA was used as a positive control. M: ladder marker. (**G**) The levels of G9a’s two protein isoforms, H3K9me2, Kcna2, and Kcna4 in the cultured DRG neurons transduced with HSV-G9a or HSV-GFP. n = 3 repeats/group. *P < 0.05 or **P < 0.01 *vs* the corresponding AAV5-GFP-treated group by two-tailed paired t-test. Full-length blots are presented in Supplemental Figure 1.

To determine how increased G9a in the axotomized DRG is involved in neuropathic pain induction, we carried out microarray analyses to identify the downstream targets of G9a in the DRG. The changes in gene expression profiles induced by G9a overexpression in the DRG were observed. The expression levels of 5,234 genes out of a total 38,851 identified genes were significantly altered in the injected DRG from the HSV-G9a-injected group compared with the HSV-GFP-injected group. Approximately 55.46% of these altered genes were downregulated and 44.54% upregulated (Fig. 5A). These altered genes are involved in multiple cell functions including immune response, cellular developmental process, tumor genesis, cell apoptosis, proliferation, migration, metabolic process, epigenetic modification, nervous system development, neurogenesis, and nerve-nerve synaptic transmission. More importantly, the expression of *Kcna2* and *Kcna4* genes was reduced by 43% and 45%, respectively, in the HSV-G9a-injected group compared to the HSV-GFP-injected group in the DRG.

Given that DRG Kcna2 is a key player in neuropathic pain genesis^4^,^10^,^11^,^12^,^20^, we examined if DRG Kcna2 is a mediator by which G9a participates in the development of neuropathic pain. We carried out quantitative real-time RT-PCR and Western blot analysis to further examine the effect of increasing DRG G9a on Kcna2 expression in the DRGs of naïve mice microinjected with HSV-G9a. G9a regulation of Kcna4 expression in DRG neurons reported previously^15^ was verified. The levels of *Ehmt2* mRNA, G9a’s two protein isoforms and H3K9me2 increased while the amounts of *Kcna2* and *Kcna4* mRNAs and proteins decreased in the injected DRGs of the HSV-G9a-injected group compared to the HSV-GFP-injected group (Fig. 5B,C). Numbers of Kcna2- and Kcna4-labeled neurons were also markedly declined in the HSV-G9a-injected DRGs compared to those in the HSV-GFP-injected DRGs (Fig. 5D). Moreover, single-cell RT-PCR revealed co-expression of *Ehmt2* mRNA with *Kcna2* and *Kcna4* mRNAs in individual small, medium and large DRG neurons (Fig. 5E,F). It is very likely that G9a directly regulates *Kcna2* and *Kcna4* expression in the DRG. Indeed, overexpression of G9a through transduction of HSV-G9a into *in vitro* cultured DRG neurons not only significantly increased the levels of G9a’s two protein isoforms as well as H3K9me2 but also reduced the amounts of Kcna2 and Kcna4 proteins (Fig. 5G).

**Figure 6:**
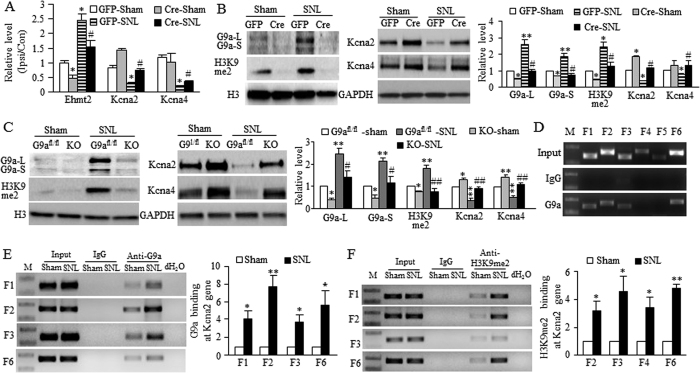
G9a is critical for nerve injury-induced downregulation of Kcna2 and Kcna4 in the axotomized DRG. (**A**,**B**) The amounts of *Ehmt2*, *Kcna2* and *Kcna4* mRNAs in the ipsilateral (Ipsi) and contralateral (Con) L4 DRG (**A**) and the levels of G9a’s two protein isoforms, H3K9me2, Kcna2, and Kcna4 in the ipsilateral L4 DRG (**B**) from the G9a^fl/fl^ mice with microinjection of AAV5-GFP or AAV5-Cre into the ipsilateral L4 DRG on day 7 post-SNL or sham surgery. n = 12 mice/group for RT-PCR and 6 mice/group for Western blots. *P < 0.05 or **P < 0.01 *vs* the AAV5-GFP-injected sham mice. ^#^*P* < 0.05 *vs* the AAV5-GFP-injected SNL mice, one-way ANOVA followed by post hoc Tukey test. Full-length blots are presented in Supplemental Figure 1. (**C**) The levels of two G9a isoforms, H3K9me2, Kcna2, and Kcna4 in the ipsilateral L4 DRG from the G9a^fl/fl^ mice and the conditional G9aKO mice on day 7 post-SNL or sham surgery. n = 12 mice/group. *P < 0.05 or **P < 0.01 *vs* the corresponding sham G9a^fl/fl^ mice. ^#^*P* < 0.05 or ^##^*P* < 0.01 *vs* the corresponding SNL G9a^fl/fl^ mice, one-way ANOVA followed by post hoc Tukey test. Full-length blots are presented in Supplemental Figure 1. (**D**) Four fragments (F1, −489/−331 bp; F2, −414/−218 bp; F3, −258/−101 bp; F6, +196/+396 bp), but not other fragments (F4, −123/+84 bp; F5, +65/+219 bp), from the promoter and 5′-end untranslated regions of the *Kcna2* gene were immunoprecipitated by the rabbit anti-G9a (not by rabbit normal IgG) in mouse DRGs. Input, total purified fragments. M, ladder marker. n = 3 repeats. (**E**) G9a bindings to F1, F2, F3, and F6 fragments within the *Kcna2* gene in the injured DRGs on day 7 post-SNL or sham surgery. n = 15 mice/group. *P < 0.05 *vs* the corresponding sham group by two-tailed paired t-test. (**F**) H3K9me2 bindings to F1, F2, F3, and F6 fragments within the *Kcna2* gene in the injured DRGs on day 7 post-SNL or sham surgery. n = 15 mice/group. *P < 0.05 *vs* the corresponding sham group by two-tailed paired t-test.

Given that peripheral nerve injury downregulates *Kcna2* and *Kcna4* in the axotomized DRG^10^,^21^ and that G9a is a gene repressor^22^, G9a may be responsible for their downregulation under neuropathic pain conditions. To this end, we determined whether blocking increased G9a in DRG affected *Kcna2* and *Kcna4* downregulation in the axotomized DRG. We microinjected AAV5-Cre into the ipsilateral L4 DRG of G9a^fl/fl^ mice to specifically and selectively block SNL-induced increases in the amounts of *Ehmt2* mRNA, G9a protein, and H3K9me2 in the axotomized DRG on day 7 post-SNL (Fig. 6A,B). AAV5-GFP was used as a control. Injection of AAV5-Cre, but not AAV5-GFP, rescued the expression of *Kcna2* and *Kcna4* mRNAs and proteins in the axotomized DRG on day 7 post-SNL (Fig. 6A,B). AAV5-Cre injection also markedly increased basal amounts of *Kcna2* mRNA and protein in the axotomized DRG of sham G9a^fl/fl^ mice on day 7 (Fig. 6A,B). Similar results were observed in the axotomized DRG of G9aKO mice on day 7 post-SNL or sham surgery (Fig. 6C). Moreover, the chromatin immunoprecipitation (ChIP) assay showed that G9a binds to four fragments (−489/−331 bp, −414/−218 bp, −258/−101 bp and +196/+396 bp) of the *Kcna2* gene as demonstrated by the amplification of only these four regions (out from 6 regions from –489 to +396 bp) from the complexes immunoprecipitated with G9a antibody in nuclear fractions from naive DRG (Fig. 6D). These binding activities were strikingly increased in the injured DRG on day 7 after SNL compared to those after sham surgery (Fig. 6E). Similar increased bindings of H3K9me2 were also observed in the injured DRG on day 7 after SNL (Fig. 6F). The evidence described above strongly suggests the contribution of G9a to nerve injury-induced *Kcna2* and *Kcna4* downregulation in the axotomized DRG.

### DRG G9a overexpression decreases total Kv current and increases excitability in DRG neurons

**Figure 7:**
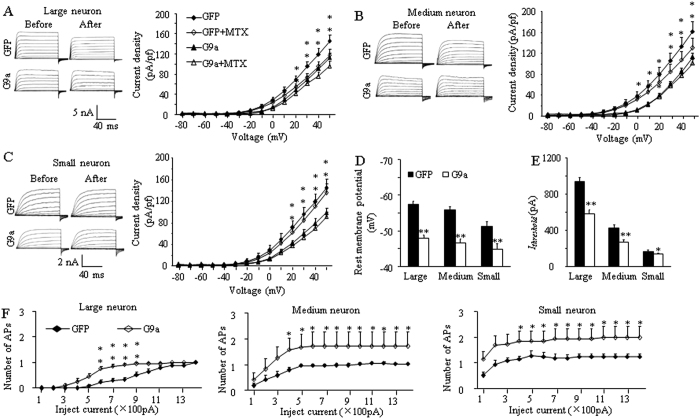
G9a overexpression reduces total Kv current and increases the excitability in the injected DRG neurons . The recording was carried out 4–7 days after microinjection of HSV-GFP (GFP) or HSV-G9a (G9a) into the unilateral L3/4 DRGs. (**A**–**C**) Representative traces of total Kv currents and I-V curves before or after bath perfusion of 100 nM maurotoxin (MTX) in the large (**A**), medium (**B**) and small (**C**) DRG neurons. n = 16 large, 17 medium, and 15 small neurons from the HSV-GFP injected group. n = 24 large, 26 medium, and 23 small neurons from the HSV-G9a-treated group. *P < 0.05 or **P < 0.01 *vs* the HSV-G9a-treated group before MTX perfusion or the HSV-GFP group after MTX perfusion, two-way ANOVA followed by post hoc Tukey test. (**D**,**E**) Resting membrane potential (**D**) and current threshold for pulses (I_threshold_, **E**). n = 28 large, 30 medium, and 21 small neurons from the HSV-GFP-treated group. n = 20 large, 19 medium, and 20 small neurons from the HSV-G9a-treated group. *P < 0.05 or **P < 0.01 *vs* the corresponding HSV-GFP-treated group, two-way ANOVA followed by post hoc Tukey test. (**F**) Numbers of evoked action potentials from the HSV-GFP- and HSV-G9a-treated groups after application of different currents. Numbers of the recorded cells are the same as in (**D**). *P < 0.05, **P < 0.01 *vs* the same stimulation intensity in the HSV-GFP-treated group, two-way ANOVA followed by post hoc Tukey test.

Since G9a reduced Kcna2 expression, we finally investigated whether mimicking the SNL-induced DRG G9a increase would affect total Kv current and the excitability in DRG neurons. Whole-cell voltage-clamp recording was carried out 4–7 days after HSV injection. Given that HSV-G9a expresses both G9a and GFP (green color), the green neurons were recorded. Total Kv current density in the HSV-G9a-injected group was significantly declined in large, medium, and small DRG neurons compared to the HSV-GFP-injected group (Fig. 7A–C). Maurotoxin (MTX) at 100 nM is a highly selective Kcna2 current inhibitor^10^,^20^,^23^,^24^. We used it to verify whether this reduction was attributed to Kcna2 downregulation. Bath application of 100 nM MTX led to a significant reduction in total Kv current in large and medium neurons from the HSV-GFP-injected group, but not from the HSV-G9a-injected group (Fig. 7A–C). When tested at +50 mV, large and medium neurons in the HSV-GFP-injected group retained 71.9% and 79.2% of current, respectively, after MTX treatment, however, both large and medium neurons from the HSV-G9a-injected group retained 86.2% and 88.3% of current, respectively. In small DRG neurons, the current reduction by MTX was not significant between HSV-GFP- and HSV-G9a-injected groups. These findings indicate that DRG G9a overexpression decreases total Kv current densities in all DRG neurons and reduces Kcna2-related current only in large and medium DRG neurons.

To examine whether DRG G9a overexpression altered DRG neuronal excitability, we carried out whole-cell current-clamp recording 4–7 days after viral injection. The resting membrane potentials in the HSV-G9a-injected group significantly increased by 9.44, 9.33, and 6.47 mV, respectively, in large, medium, and small neurons compared to the HSV-GFP-injected group (Fig. 7D). The current thresholds in the HSV-G9a-injected group decreased by 37.7%, 37.1%, and 18.7% of the values in the HSV-GFP-injected group in large, medium, and small neurons, respectively (Fig. 7E). The average number of action potentials (APs) evoked by stimulation of between 600 and 900 pA in the HSV-G9a-injected group was greater than that in the HSV-GFP-injected group in large neurons, whereas the stimulation of ≥400 pA in the HSV-G9a-injected group was greater than that in the HSV-GFP-injected group in medium and small neurons (Fig. 7F). There are no significant changes in other AP parameters such as membrane input resistance, AP threshold, AP overshoot, AP amplitude and after hyperpolarization amplitude (Table 1). These data indicate that DRG G9a overexpression increases DRG neuronal excitability.

## Discussion

This study provides the first evidence to our knowledge that nerve injury-induced increases in G9a and its catalyzed repressive marker H3K9me2 are responsible for epigenetic silencing of *Kcna2* in the axotomized DRG neurons. Mimicking these increases reduces Kcna2 expression and total Kv current and increases excitability in the DRG neurons and leads to pain hypersensitivity. Conversely, blocking these increases rescues Kcna2 expression in the axotomized DRG and attenuates nerve injury-induced pain hypersensitivities. DRG G9a contributes to neuropathic pain likely by silencing *Kcna2* expression in the axotomized DRG.

G9a, a major euchromatic methyltransferase, is widely expressed in most tissues, including the peripheral nervous system^25^,^26^,^27^. The human and mouse G9a proteins have two alternatively spliced isoforms, long and short^13^,^22^, which have been identified in mouse DRG and spinal cord in the present study and in other tissues in previous work^28^,^29^,^30^. However, a recent study did not detect these two protein isoforms in the DRG tissue^15^. The reason for this difference between the present and previous studies is unknown but may be related to distinct primary antibodies used. Although G9a’s two protein isoforms are expressed at a low level in normal DRG, they can be activated at the transcriptional level. Peripheral nerve injury increased expression of *Ehmt2* mRNA and G9a’s two protein isoforms as well as H3K9me2 in the axotomized DRG, but not intact DRG and spinal cord. Interestingly, CFA-induced peripheral inflammation did not alter levels of G9a’s two protein isoforms and H3K9me2 in either DRG or spinal cord. It appears that G9a transactivation in DRG is tissue- and nerve injury-specific. Our double-labeling immunohistochemistry further demonstrated that this transactivation occurs exclusively in DRG neurons.

Peripheral nerve injury alters the expression of genes encoding receptors, enzymes, and ion channels in the axotomized DRG^12^,^31^,^32^. G9a may be a key regulator of these changes under neuropathic pain conditions. Laumet *et al*. reported that G9a inhibition restored 396 genes downregulated by nerve injury and normalized 242 genes upregulated by nerve injury in the axotomized DRG^15^. They further demonstrated that G9a was required for nerve injury-induced silencing of *Kcna4, Kcnd2, Kcnq2*, and *Kcnma1* genes in the DRG^15^. However, no direct evidence demonstrated the participation of these target K^+^ channels in neuropathic pain due to the lack of selective and specific antagonists/inhibitors for these K^+^ channels. Thus, whether G9a contributes to neuropathic pain through the silencing of these K^+^ channels in the DRG neurons is still uncertain. Our gene microarray analysis showed that DRG G9a overexpression altered the expression of 5,234 genes, in which 2,903 genes were downregulated and 2,331 genes upregulated. More importantly, our microarray assay revealed that overexpression of G9a significantly reduced *Kcna2* RNA expression in the DRG, which was further confirmed by our quantitative RT-PCR and Western blot assays. Although the contribution of most of these altered genes to neuropathic pain was unclear, DRG Kcna2 is a key player in neuropathic pain. Rescuing Kcna2 downregulation by overexpressing *Kcna2* RNA or blocking increased *Kcna2* AS RNA in the axotomized DRG ameliorated neuropathic pain^4^,^10^,^20^. Mimicking nerve injury-induced Kcna2 downregulation by overexpressing *Kcna2* AS RNA in DRG reduced total Kv current, depolarized resting membrane potential, decreased current threshold for inducing AP, and increased the excitability in the DRG neurons and led to neuropathic pain-like behaviors^4^,^10^,^20^. Present data showed that blocking increased G9a rescued Kcna2 expression in the axotomized DRG and attenuated neuropathic pain. G9a overexpression in the DRG led to similar phenomena to those produced by DRG *Kcna2* AS RNA overexpression. Kcna2 belongs to a family of delayed rectifiers, which allow DRG neurons to efficiently repolarize following an action potential. How *Kcna2* downregulation induced by overexpression of either G9a or *Kcna2* AS RNA participates in resting membrane depolarization in the DRG neurons is still unclear. The changes in the expression of other channels induced directly by G9a overexpression and/or following *Kcna2* downregulation in the DRG neurons may be involved in resting membrane depolarization observed presently. Given that G9a is co-localized with *Kcna2* in the DRG neurons, it is very likely that G9a participates in the mechanisms underlying neuropathic pain development by downregulating *Kcna2* in the axotomized DRG. However, other potential mechanisms of G9a involvement in neuropathic pain cannot be ruled out, because G9a regulates the expression of other genes in the axotomized DRG^33^.

It should be noted that multiple mechanisms are involved in nerve injury-induced Kcna2 silencing in the axotomized DRG. In addition to G9a’s role revealed in the present study and *Kcna2* AS RNA reported previously^10^, our recent work showed that DNMT3a-triggered *de novo* DNA methylation in the promoter and 5′-untranslated regions of the *Kcna2* gene was crucial in nerve injury-induced Kcna2 silencing in the axotomized DRG (data not shown). How these three epigenetic mechanisms work together to regulate Kcna2 expression and whether they interact with/affect each other under neuropathic pain conditions are unknown and remain to be further investigated. In addition, a decrease in Kcna2 mRNA levels may be the result of decreased mRNA stability and/or reduced transcription factors-triggered transcriptional activation. These potential possibilities will also be addressed in our future studies.

In summary, our study revealed a G9a-triggered epigenetic mechanism of how *Kcna2* is downregulated in the axotomized DRG under neuropathic pain conditions. Given that DRG G9a inhibition or knockout produces antinociceptive effects without affecting motor function and basal pain perception, G9a could be a novel target in neuropathic pain management.

## Materials and Methods

### Animal preparations

C57BL/6 J wild-type mice, G9a^fl/fl^ mice^34^, and Advillin^Cre/+^ mice^35^ were used in this study. G9a^fl/fl^ mice were fully backcrossed to C57BL/6 J mice and were homozygous for a floxed G9a allele. Male sensory-specific Cre line Advillin^Cre/+^ mice were crossed with female G9a^fl/fl^ mice to obtain G9a conditional knockout (G9aKO) mice. All animals were kept in a standard 12-h light/dark cycle, with water and food pellets available *ad libitum*. Male mice weighing 25–30 g were used for behavior testing. All procedures used were approved by the Animal Care and Use Committee at Rutgers New Jersey Medical School and are consistent with the ethical guidelines of the US National Institutes of Health and the International Association for the Study of Pain. All efforts were made to minimize animal suffering and to reduce the number of animals used. All of the experimenters were blind to treatment condition.

### DRG microinjection

DRG microinjection was carried out as described^10^,^20^ with minor modification. Briefly, a midline incision was made in the lower lumbar back region, and the L_3_ and/or L_4_ articular processes were exposed and then removed with small ronguers. After the DRG was exposed, viral solution (0.5 μl) was injected into two sites in the L_3_ and L_4_ DRGs or into one site in the L_4_ DRG with a glass micropipette connected to a Hamilton syringe. The pipette was removed 10 min after injection. The surgical field was irrigated with sterile saline and the skin incision closed with wound clips. The injected mice displayed no sign of paresis or other abnormalities. The immune responses from viral injection were thus minimal. AAV5-GFP and AAV5-Cre were purchased from UNC Vector Core (Chapel Hill, NC). HSV-GFP and HSV-G9a (expressing both G9a and GFP) were provided by Dr. Eric J Nestler^34^.

### Neuropathic pain models

L_4_ Spinal nerve ligation (SNL) and chronic constriction injury of sciatic nerve (CCI) were carried out as described previously^10^,^20^. Briefly, SNL was performed by ligation of the fourth lumbar spinal nerve with 7–0 silk suture and transection at the distal site. CCI was done loosely ligation of sciatic nerve with 4-0 chromic gut thread at 4 sites with an interval of 1 mm. Sham-operative groups underwent identical procedures but without ligation of the respective nerve. Mechanical, thermal, and cold behavioral tests as described below were performed before surgery (−35 and −1 days) and 3, 5, 7, 10, 12, or 14 days after surgery.

### Behavioral tests

Mechanical, thermal, and cold behavioral tests were carried out as described^10^,^36^. These behavioral tests were conducted at 1 hour intervals. Paw withdrawal frequencies in response to mechanical stimuli (calibrated von Frey filaments) were first measured. Paw withdrawal latencies to noxious heat were then measured with a Model 336 Analgesia Meter (IITC Inc. Life Science Instruments. Woodland Hills, CA). Finally, paw withdrawal latencies to noxious cold (0 °C) were measured with a cold aluminum plate.

### DRG neuronal culture and transfection

Primary DRG neuronal cultures and viral transfection were carried out as described^10^. Briefly, adult mice were euthanized with isoflurane and all DRGs were collected in cold Neurobasal Medium (Gibco/ThermoFisher Scientific) with 10% fetal bovine serum (JR Scientific, Woodland, CA), 100 units/ml Penicillin, 100 μg/ml Streptomycin (Quality Biological, Gaithersburg, MD) and then treated with enzyme solution (5 mg/ml dispase, 1 mg/ml collagenase type I in Hanks’ balanced salt solution (HBSS) without Ca^2+^ and Mg^2+^ (Gibco/ThermoFisher Scientific)). After trituration and centrifugation, dissociated cells were resuspended in mixed Neurobasal Medium and plated in a six-well plate coated with 50 μg/ml poly-D-lysine (Sigma, St. Louis, MO). The cells were incubated at 95% O_2_, 5% CO_2_, and 37 °C. One day later, 1 μl of virus (titer ≥1 × 10^12^/ml) was added to each 2 ml-well. Neurons were collected 2 days later.

### Reverse transcription (RT)-polymerase chain reaction (PCR)

For quantitative real-time RT-PCR, four unilateral mouse DRGs were pooled together to achieve enough RNA. Total RNA was extracted by the Trizol method (Invitrogen/ThermoFisher Scientific), treated with DNase I (New England Biolabs, Ipswich, MA), and reverse-transcribed using the ThermoScript reverse transcriptase (Invitrogen/ThermoFisher Scientific), random hexamers, oligo (dT) primers or specific RT-primers (Table 2). The reverse primer of each pairs of primers were used as RT-primers. Template (1 μl) was amplified by real-time PCR by using the primers listed in the Table 2 (Integrated DNA Technologies). GAPDH was used as an internal control for normalization. Each sample was run in triplicate in a 20 μL reaction with 250 nM forward and reverse primers, 10 μl of SsoAdvanced Universal SYBR Green Supermix (Bio-Rad Laboratories, Hercules, CA) and 20 ng of cDNA. Reactions were performed in a BIO-RAD CFX96 real-time PCR system. Ratios of ipsilateral-side mRNA levels to contralateral-side mRNA levels were calculated using the ΔCt method (2^−ΔΔCt^). All data were normalized to GAPDH, which has been demonstrated to be stable even after peripheral nerve injury insult^10^.

For single-cell real-time RT-PCR, freshly dissociated mouse DRG neurons were first prepared as described previously^10^. Briefly, four hours after plating, a single living DRG neuron was collected under an inverted microscope fit with a micromanipulator and microinjector and placed in a PCR tube with 5–10 μl of cell lysis buffer (Signosis, Sunnyvale, CA). After centrifugation, the supernatants were collected. The remaining real-time RT-PCR procedure was carried out according to the manufacturer’s instructions with the single-cell real-time RT-PCR assay kit (Signosis). All primers used are listed in Table 2.

### Single- or double-labeled immunohistochemistry

Mice were anesthetized with isoflurane and perfused with 4% paraformaldehyde before being analyzed by single- or double-labeled immunohistochemistry. L3 and L4 DRGs were removed, post-fixed, and dehydrated before frozen sectioning at 20 μm. After the sections were blocked for 1 h at room temperature in 0.01 M PBS containing 10% goat serum and 0.3% Triton X-100, they were incubated with the following primary antibodies or the regents over one or two nights at 4 °C. The antibodies and regents include: rabbit anti-G9a (1:20, Abcam, Cambridge, MA), rabbit anti-H3K9me2 (H3K9; 1:50, EMD Millipore, Darmstadt, Germany), mouse anti-NF200 (1:500, Sigma), biotinylated IB4 (1:100, Sigma), mouse anti-CGRP (1:50, Abcam), mouse anti-NeuN (1:50, GeneTex, Irvine, CA), mouse anti-glutamine synthetase (GS; 1:500, EMD Millipore), rabbit anti-Kv1.2 (1:200, Alomone labs, Jerusalem, Israel), and rabbit anti-Kv1.4 (1:200, EMD Millipore). The sections were then incubated with either goat anti-rabbit antibody conjugated to Cy3 (1:200, Jackson ImmunoResearch, West Grove, PA), and/or monkey anti-mouse antibody conjugated to Cy2 (1:200, Jackson ImmunoResearch), or avidin labeled with FITC (1:200, Sigma) for 2 h at room temperature. Control experiments included substitution of normal mouse or rabbit serum for the primary antiserum and omission of the primary antiserum. All immunofluorescence-labeled images were examined using a Leica DMI4000 fluorescence microscope and captured with a DFC365FX camera (Leica, Germany). Single- or double-labeled neurons were quantified manually or by using NIH Image J Software.

### Western blotting

To achieve enough proteins, four unilateral mouse DRGs were pooled together. Tissues were homogenized and the cultured cells ultrasonicated in chilled lysis buffer (10 mM Tris, 1 mM phenylmethylsulfonyl fluoride, 5 mM MgCl_2_, 5 mM EGTA, 1 mM EDTA, 1 mM DTT, 40 μM leupeptin, 250 mM sucrose). After centrifugation at 4 °C for 15 min at 1,000 *g*, the supernatant was collected for cytosolic proteins and the pellet for nuclear proteins. The contents of the proteins in the samples were measured using the Bio-Rad protein assay (Bio-Rad) and then the samples were heated at 99 °C for 5 min and loaded onto a 4–15% stacking/7.5% separating SDS-polyacrylamide gel (Bio-Rad Laboratories). The proteins were then electrophoretically transferred onto a polyvinylidene difluoride membrane (Bio-Rad Laboratories). After the membranes were blocked with 3% nonfat milk in Tris-buffered saline containing 0.1% Tween-20 for 1 h, the following primary antibodies were used: mouse anti-Kv1.2 (1:500, NeuroMab, Davis, CA), mouse anti-Kv1.4 (1:500, NeuroMab), rabbit anti-GAPDH (1:1000, Santa Cruz, Dallas, Texas), mouse anti-α-tublin (1:1000, Santa Cruz), rabbit anti-G9a (1:1000, Cell signaling, Danvers, MA), rabbit anti-H3K9me2 (1:500, EMD Millipore), rabbit anti-phospho-ERK1/2 (Thr202/Tyr204, 1:1,000, Cell Signaling), rabbit anti-ERK1/2 (1:1,000, Cell Signaling), mouse anti-GFAP (1:1,000, Cell Signaling), and rabbit anti-histone H3 (1:1,000, Cell Signaling), The proteins were detected by horseradish peroxidase–conjugated anti-mouse or anti-rabbit secondary antibody (1:3,000, Jackson ImmunoResearch) and visualized by western peroxide reagent and luminol/enhancer reagent (Clarity Western ECL Substrate, Bio-Rad) and exposure by ChemiDoc XRS and System with Image Lab software (Bio-Rad). The intensity of blots was quantified with densitometry using Image Lab software (Bio-Rad). All cytosol protein bands were normalized to either α-tubulin or GAPDH, whereas all nucleus protein to total histone H3.

### Chromatin immunoprecipitation (ChIP)

The ChIP assays were conducted using the EZ ChIP Kit (Upstate/EMD Millipore, Darmstadt, Germany) as described^10^. The homogenization solution from DRGs was crosslinked with 1% formaldehyde for 10 min at room temperature. The reaction was terminated by the addition of 0.25 M glycine. After centrifugation, the collected pellet was lysed by SDS lysis buffer with protease inhibitor cocktail and sonicated until the DNA was broken into fragments with a mean length of 200 to 1,000 bp. After the samples were pre-cleaned with protein G agarose, they were subjected to immunoprecipitation overnight with 2 μg of rabbit antibodies against G9a (Abcam) and H3K9me2 (Abcam), or with 2 μg of normal rabbit serum overnight at 4 °C. Input (10–20% of the sample for immunoprecipitation) was used as a positive control. The DNA fragments were purified and identified using PCR/Real-time PCR with the primers listed in Table 2.

### Gene microarray

Two groups (3 repeats/group), G9a-injected and GFP-injected groups, were carried out. Briefly, 4 days after microinjection of HSV-G9a or HSV-GFP into unilateral L3 sand L4 DRGs, mice were rapidly decapitated and the injected DRGs were removed, quickly frozen in liquid nitrogen, and then transferred to −80 °C until RNA was extracted. Injected DRGs from 2 mice were pooled together. Total RNA was extracted by the Trizol method (Invitrogen) and treated with excess DNase I (New England Biolabs, Ipswich, MA). RNA quality was checked using Agilent’s Bioanalyzer. Reverse transcription, amplification, labeling and hybridization to Affymetrix Mouse Exon 1.0ST arrays were performed using standard procedures at Johns Hopkins Deep Sequencing & Microarray Core Facility. Raw data were extracted from the raw Affymetrix CEL files using Partek Genomics Suite 6.6 platform (Partek Inc., St. Louis MO, USA). The extended gene transcript set was imported and normalized using the RMA (Robust Multiarray Analysis) algorithm, then compared between two groups using a one-way ANOVA. Normalized data were further analyzed using Spotfire DecisionSite for Functional Genomics software (TIBCO Spotfire, Boston MA, USA). Log_2_ ratios representing the difference in the expression level were obtained on the basis of the expression level from the GFP-injected group. Gene lists were generated using significance criteria of a linear 1.2 fold change cutoff coupled with a nonstringent p-value cutoff of *P* < 0.05, more than 5 probes per transcript and appropriate genomic mapping. Evaluation of lists of differentially expressed genes for enrichment in predefined categories and functional groups of genes was carried out using functional analysis tools GORILLA. To minimize batch effects and maintain a high degree of confidence in these data, all animals were handled, treated and euthanized at the same time under the same conditions and the processing of all RNAs and arrays carried out at the same time.

### Whole-cell patch clamp recording

To record total potassium current in DRG neurons, we first prepared dissociated mouse L3/L4 DRG neurons as described above. Whole-cell patch clamp recording was carried out 4 to 8 h after plating. To improve recording efficiency, only green labeled neurons were recorded. Coverslips were placed in the perfusion chamber (Warner Instruments, Hamden, CT). The electrode resistances of micropipettes ranged from 3 to 5 MΩ. Neurons were voltage-clamped with an Axopatch-700B amplifier (Molecular Devices, Sunnyvale, CA). The intracellular pipette solution contained (in mM) potassium gluconate 120, KCl 20, MgCl_2_ 2, EGTA 10, HEPES 10, and Mg-ATP 4 (pH 7.3 with KOH, 310 mOsm). We minimized the Na^+^ and Ca^2+^ component in voltage-gated potassium current recording using an extracellular solution composed of (in mM) choline chloride 150, KCl 5, CdCl_2_ 1, CaCl_2_ 2, MgCl_2_ 1, HEPES 10, glucose 10 (pH 7.4 with Tris base, 320 mOsm). Signals were filtered at 1 kHz and digitized by using a DigiData 1550 with pClamp 10.4 software (Molecular Devices). Series resistance was compensated by 60–80%. Cell membrane capacitances were acquired by reading the value for whole-cell capacitance compensation directly from the amplifier. An online P/4 leak subtraction was performed to eliminate leak current contribution. The data were stored on a computer by a DigiData 1550 interface and were analyzed by the pCLAMP 10.4 software package (Molecular Devices).

To record the action potential, we switched the recording mode into current clamp. The extracellular solution consisted of (in mM) NaCl 140, KCl 4, CaCl_2_ 2, MgCl_2_ 2, HEPES 10 and glucose 5, with pH adjusted to 7.38 by NaOH. The intracellular pipette solution contained (in mM) KCl 135, Mg-ATP 3, Na_2_ATP 0.5, CaCl_2_ 1.1, EGTA 2 and glucose 5; pH was adjusted to 7.38 with KOH and osmolarity adjusted to 300 mOsm with sucrose. The resting membrane potential was taken 3 min after a stable recording was first obtained. Depolarizing currents of 100–1,400 pA (200-ms duration) were delivered in increments of 100 pA until an action potential (AP) was evoked. The injection current threshold was defined as the minimum current required to evoke the first AP. The membrane potential was held at the existing resting membrane potential during the current injection. The AP threshold was defined as the first point on the rapid rising phase of the spike at which the change in voltage exceeded 50 mV/ms. The AP amplitude was measured between the peak and the baseline. The membrane input resistance for each cell was obtained from the slope of a steady-state I–V plot in response to a series of hyperpolarizing currents, 200-ms duration delivered in steps of 100 pA from 200 pA to −2,000 pA. The after-hyperpolarization amplitude was measured between the maximum hyperpolarization and the final plateau voltage, and the AP overshoot was measured between the AP peak and 0 mV. The data were stored on a computer by a DigiData 1550 interface and were analyzed by the pCLAMP 10.4 software package (Molecular Devices). All experiments were performed at room temperature.

### Statistical analysis

For *in vitro* experiments, the cells were evenly suspended and then randomly distributed in each well tested. For *in vivo* experiments, the animals were distributed into various treatment groups randomly. All of the results are given as means ± S.E.M. The data were statistically analyzed with two-tailed, paired Student’s *t*-test and a one-way or two-way ANOVA. When ANOVA showed a significant difference, pairwise comparisons between means were tested by the *post hoc* Tukey method (SigmaPlot 12.5, San Jose, CA). Significance was set at *P* < 0.05.

## Additional Information

**How to cite this article**: Liang, L. *et al*. G9a participates in nerve injury-induced Kcna2 downregulation in primary sensory neurons. *Sci. Rep*. **6**, 37704; doi: 10.1038/srep37704 (2016).

**Publisher’s note:** Springer Nature remains neutral with regard to jurisdictional claims in published maps and institutional affiliations.

## Supplementary Material

Supplementary Information

## Figures and Tables

**Table 1:** Membrane input resistance and other action potential parameters in DRG neurons.

	Large neuron		
	HSV-GFP	HSV-G9a	t/p value
**n**	**28 cells, 6 mice**	**20 cells, 5 mice**	
Rin, MΩ	76.24 ± 14.39	86.51 ± 6.54	−0.531/0.598
APT, mV	−16.50 ± 0.91	−16.40 ± 1.15	0.068/0.946
APO, mV	37.83 ± 2.18	43.67 ± 2.78	1.596/0.118
APA, mV	89.20 ± 2.35	95.19 ± 2.40	1.691/0.098
AHPA, mV	−12.59 ± 0.76	−14.21 ± 0.52	1.625/0.111
	**Medium neuron**		
	**HSV-GFP**	**HSV-G9a**	**t/p value**
**n**	**30 cells, 6 mice**	**19 cells, 5 mice**	
Rin, MΩ	161.77 ± 17.23	179.70 ± 17.69	−0.681/0.500
APT, mV	−16.10 ± 1.11	−14.03 ± 0.76	−1.326/0.193
APO, mV	40.65 ± 2.88	43.98 ± 2.16	−0. 829/0.411
APA, mV	95.24 ± 3.21	92.56 ± 3.16	0.563/0.576
AHPA, mV	−16.09 ± 1.10	−18.05 ± 1.21	−1.168/0.249
	**Small neuron**		
	**HSV-GFP**	**HSV-G9a**	**t/p value**
**n**	**21 cells, 6 mice**	**20 cells, 5 mice**	
Rin, MΩ	216.20 ± 14.77	217.36 ± 28.40	−1.396/0.171
APT, mV	−17.90 ± 1.41	−14.83 ± 0.97	1.841/0.074
APO, mV	36.80 ± 4.77	41.97 ± 1.96	−1.037/0.306
APA, mV	85.40 ± 4.54	90.06 ± 2.41	−0.926/0.360
AHPA, mV	−15.20 ± 1.78	−19.14 ± 1.55	1.719/0.094

Values are Mean ± S.E.M., Rin: membrane input resistance. APT: action potential threshold. APA: action potential amplitude. APO: action potential overshoot. AHPA: after hyperpolarization amplitude.

**Table 2:** All primers used.

Names	Sequences
Real-time RT-PCR	
Ehmt2-F	5′-AAATTGGGAACTTGGAAATGG-3′
Ehmt2-R	5′-CACTACCCGTGAAGGAGGC-3′
Kcna2-RT	5′-GTCCCCGTCACATCTTCTCACT-3′
Kcna2-F	5′-CTGCAAGGGCAACGTCACAC-3′
Kcna2-R	5′-GGGACAGTGAGATGCTTGGC-3′
Kcna4-RT	5′-GGATGCTGTCCGGTGATGAC-3′
Kcna4-F	5′-TGCTGGGAATGGTGAAGTGC-3′
Kcna4-R	5′-CCCACAGACAATGCCAGGTT-3′
Gapdh-RT	5′-TCGTGGTTCACACCCATCAC-3′
Gapdh-F	5′-TCGGTGTGAACGGATTTGGC-3′
Gapdh-R	5′-TCCCATTCTCGGCCTTGACT-3′
ChIP	
F1-F	5′-ATTGTCCTGGGAAACCGAGT-3′
F1-R	5′-CCGAGGGAGATGTGTTGCTA-3′
F2-F	5′-AGAAGCAGAAGGCAGGAGTG-3′
F2-R	5′-GGAGACAGGGGAGAGGGTAG-3′
F3-F	5′-CTCTCAAGCGCTCCTCAACT-3′
F3-R	5′-TGGGTTAGATCCCTGTGTCC-3′
F4-F	5′-CCGGGACACAGGGATCTAAC-3′
F4-R	5′-GGGAGCCGACTCTGCAGT-3′
F5-F	5′-GGACTGCAGAGTCGGCTC-3′
F5-R	5′-CAGCAGAATGCCGGACACT-3′
F6-F	5′-AAATCAGTGTCCGGCATTCT-3′
F6-R	5′-CCCATTCCATGCACTCTTCT-3′

RT: Reverse-transcription. F: Forward. R: Reverse.
