# Supplementary material for: G9a participates in nerve injury-induced Kcna2 downregulation in primary sensory neurons
Source: Sci Rep. 2016 Nov 22;6:37704. doi: 10.1038/srep37704 (PMC5118693; doi:10.1038/srep37704)

Title: G9a participates in nerve injury-induced Kcna2 downregulation in primary sensory neurons

Authors: Lingli Liang[1],* Xiyao Gu[1],* Jian-Yuan Zhao[1,2],* Shaogen Wu[1], Xuerong Miao[1], Jifang Xiao[1], Kai Mo[1], Jun Zhang[1], Brianna Marie Lutz[1], Alex Bekker[1], Yuan-Xiang Tao[1,3]

Supplementary Fig. 1 Full-length pictures of the blots presented in the main figures

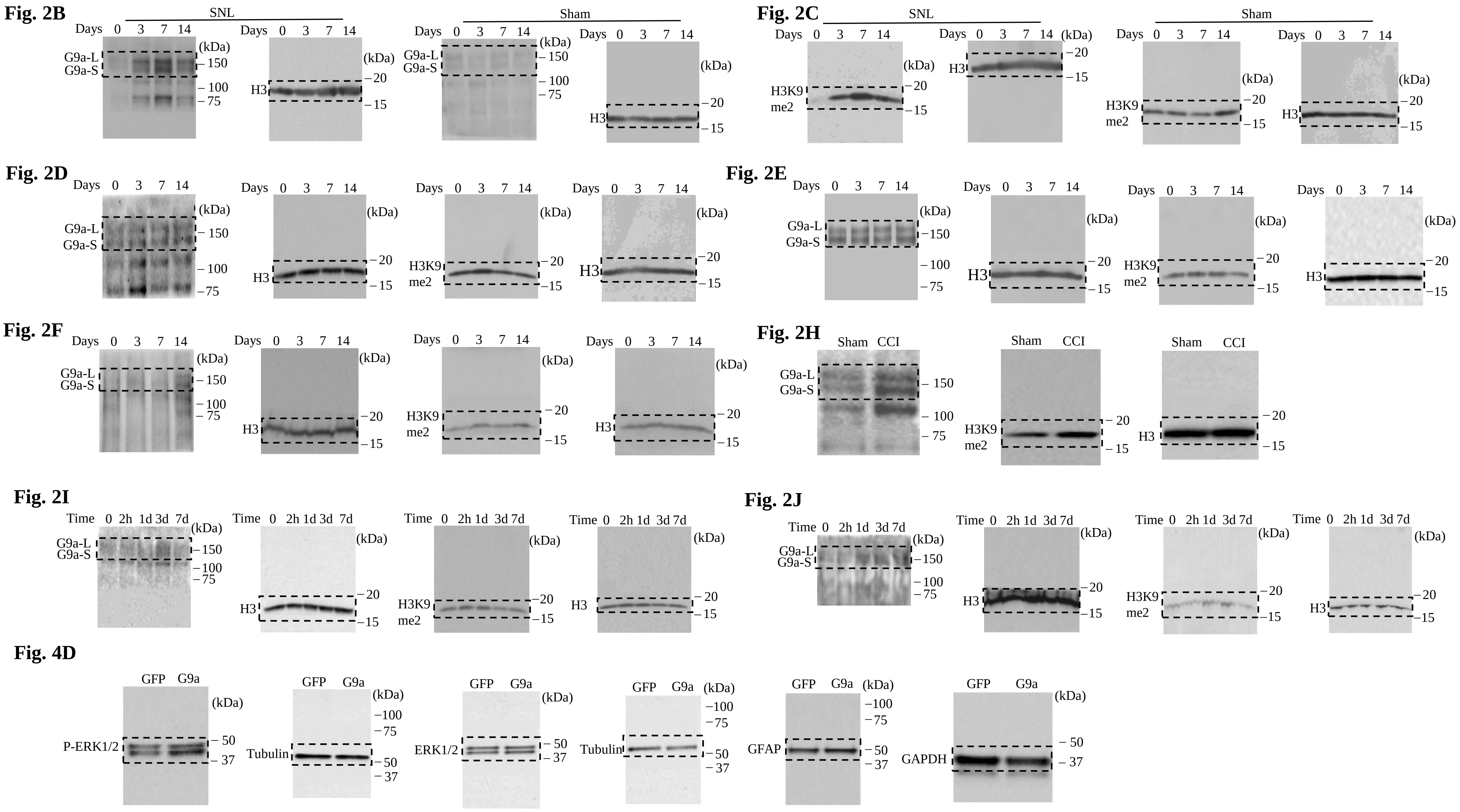

Fig. 5C

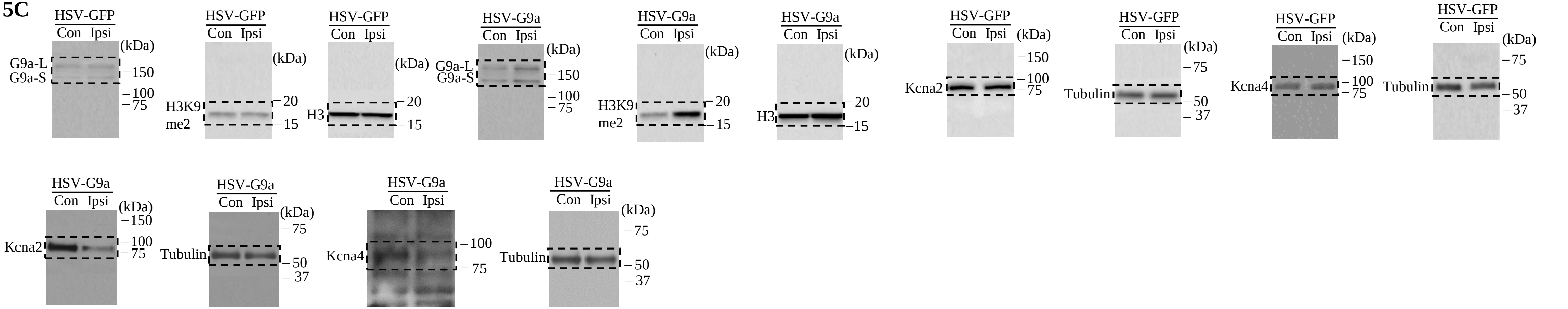

Fig. 5G

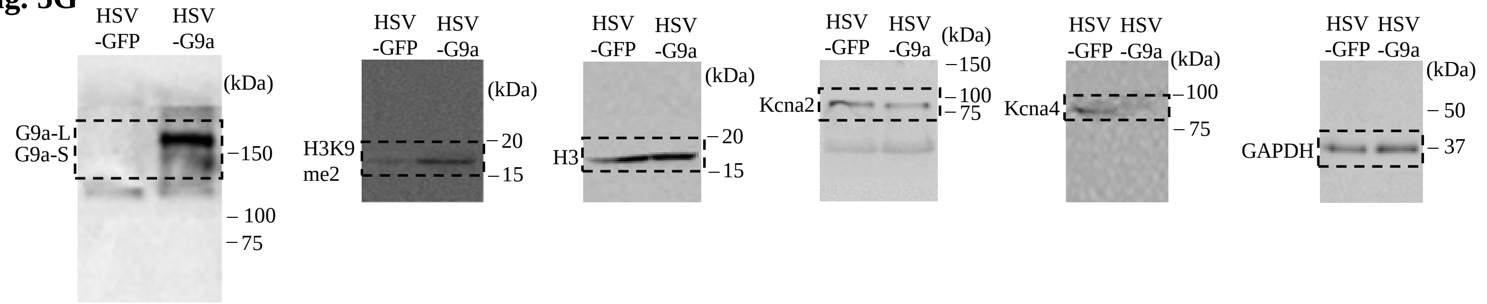

Fig. 6B

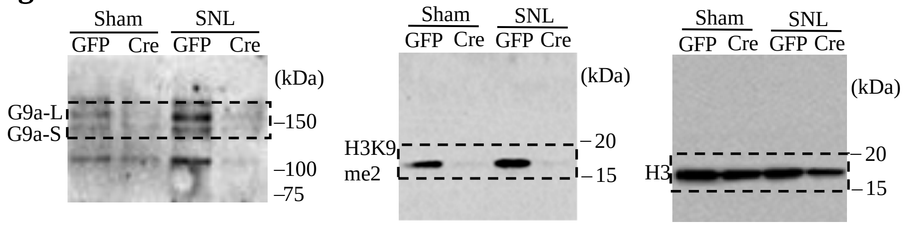

Fig. 6B

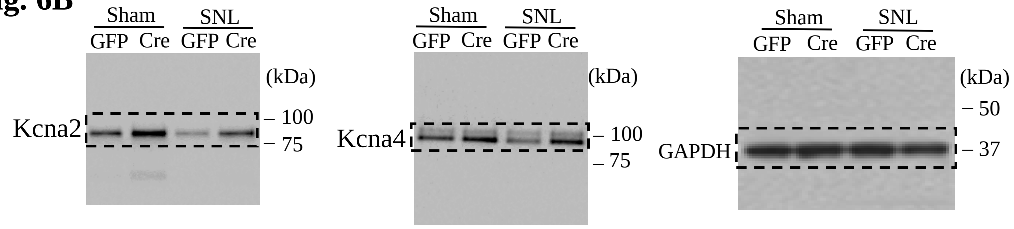

Fig. 6C

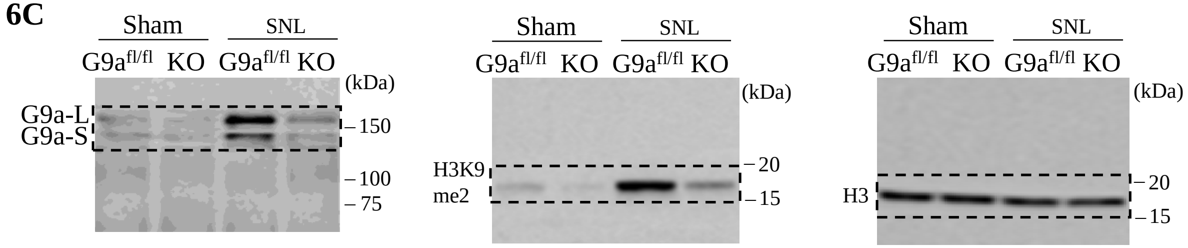

Fig. 6C

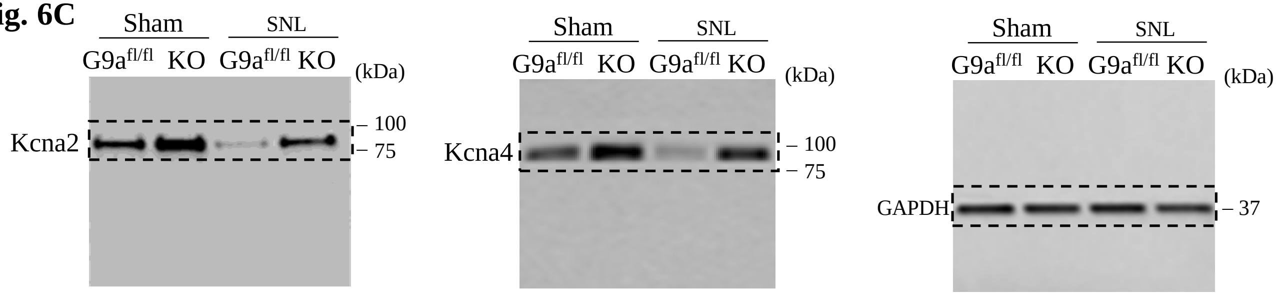

Supplement: Supplementary Information [file srep37704-s1.pdf]
